# A novel and rapid method for obtaining high titre intact prion strains from mammalian brain

**DOI:** 10.1038/srep10062

**Published:** 2015-05-07

**Authors:** Adam Wenborn, Cassandra Terry, Nathalie Gros, Susan Joiner, Laura D’Castro, Silvia Panico, Jessica Sells, Sabrina Cronier, Jacqueline M. Linehan, Sebastian Brandner, Helen R. Saibil, John Collinge, Jonathan D. F. Wadsworth

**Affiliations:** 1 MRC Prion Unit and Department of Neurodegenerative Disease, UCL Institute of Neurology, National Hospital for Neurology and Neurosurgery, Queen Square, London WC1N 3BG, UK; 2 Department of Crystallography and Institute of Structural and Molecular Biology, Birkbeck College, University of London, Malet Street, London WC1E 7HX, UK

## Abstract

Mammalian prions exist as multiple strains which produce characteristic and highly reproducible phenotypes in defined hosts. How this strain diversity is encoded by a protein-only agent remains one of the most interesting and challenging questions in biology with wide relevance to understanding other diseases involving the aggregation or polymerisation of misfolded host proteins. Progress in understanding mammalian prion strains has however been severely limited by the complexity and variability of the methods used for their isolation from infected tissue and no high resolution structures have yet been reported. Using high-throughput cell-based prion bioassay to re-examine prion purification from first principles we now report the isolation of prion strains to exceptional levels of purity from small quantities of infected brain and demonstrate faithful retention of biological and biochemical strain properties. The method’s effectiveness and simplicity should facilitate its wide application and expedite structural studies of prions.

Prions are lethal infectious agents that cause fatal neurodegenerative diseases in mammals, including Creutzfeldt-Jakob disease (CJD) in humans, scrapie in sheep and goats and bovine spongiform encephalopathy (BSE) in cattle^1^,^2^. They are unique pathogens (being devoid of significant coding nucleic acid) and are composed of infectious polymeric assemblies of misfolded host-encoded cellular prion protein (PrP^C^), some of which acquire protease-resistance and are classically designated as PrP^Sc^^1^,^2^. Prions propagate by means of seeded protein polymerization, a process that involves the binding and templated misfolding of PrP^C^ followed by fission of the polymer to produce more seeds. Different prion strains can propagate in the same inbred host expressing identical PrP^C^ to produce different disease phenotypes and appear to be encoded by distinct PrP conformations and assembly states^1^,^2^,^3^,^4^,^5^,^6^. Many other proteins are also capable of generating self-propagating polymeric or amyloid conformations and, although these lack the overt infectivity of prions, seeded protein misfolding is generically relevant in the pathogenesis of many other human diseases, including Alzheimer’s and Parkinson’s disease^7^,^8^,^9^.

Despite such advances, structural analysis of mammalian prions at a resolution sufficient to differentiate them from non-infectious fibrillar PrP assemblies has not yet been achieved^10^,^11^. This has been prevented by two central problems. Firstly the difficulty of isolating relatively homogeneous prion particles from affected tissue and unequivocally correlating infectivity with composition and structure, and secondly, an inability to systematically produce high-titre synthetic prions from fully defined starting materials. Although the production of prions *in vitro* from recombinant PrP (either alone or in combination with non-protein cofactors) has been reported, no preparation as yet has been shown to have a prion titre that is compatible with meaningful structural analysis^2^,^10^. Consequently, the isolation of *ex vivo* prions with high specific infectivity in a form suitable for structural study remains a key goal.

Purification of prions from mammalian brain is a formidable problem, hindered principally by their low abundance in affected tissue and the requirement to maintain and measure biological activity throughout purification. The seminal purification procedures developed by Prusiner and colleagues^12^,^13^,^14^,^15^,^16^ were a major accomplishment and have remained the benchmark in the field for the last thirty years. However the technical complexity of these procedures and requirement for large quantities of infected brain tissue has limited their widespread application. Concomitantly, exploration of alternative purification strategies has been severely restricted by an historical reliance on expensive, very time-consuming and rather imprecise rodent bioassays.

The development of cell culture-based prion methods^17^, and their automation at the MRC Prion Unit, has been revolutionary and has allowed highly precise and reproducible prion assays^18^,^19^ and an entirely fresh approach to prion purification, with rapid iterative assay of all fractions for prion infectivity unbiased by previous published experience. Here we report the use of cell culture based prion bioassay to develop a rapid and simple protocol for isolating highly purified prions from mammalian brain. We show that the method works equally well with different prion strains from different hosts and yields extremely high-titre infectious prion preparations that contain full-length PrP of exceptional purity. As these methods utilise reagents that are readily commercially available and do not require specialised equipment they should be practicable in most existing prion laboratories.

## Results

### Method development

**Figure 1:**
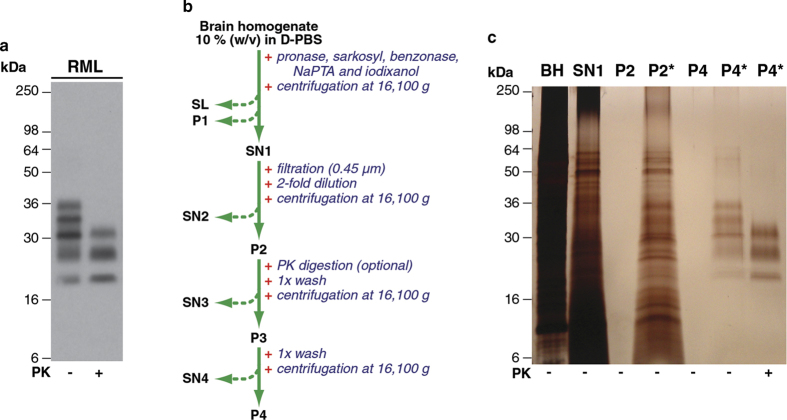
Overview of the purification method. (**a**) Western blot of 10% (w/v) terminal RML-infected CD1 mouse brain homogenate before (-) or after (+) digestion with proteinase K (PK) using anti-PrP monoclonal antibody ICSM35. (**b**) Purification scheme (SL, surface layer; P, pellet, SN, supernatant). (**c**) Silver-stained 16% SDS-PAGE gel showing RML-infected CD1 mouse brain homogenate (BH) and purified fractions. The equivalent of 2 μl or 200 μl (denoted by *) of 10% (w/v) brain homogenate was loaded per lane. +,– symbols indicate whether PK-digestion was applied during purification (to the P2 sample, see panel b).

Using the Scrapie Cell Assay (SCA) for precise and high-throughput *in vitro* cell-based prion bioassay^17^,^20^,^21^ we systematically investigated new methods for purifying RML prions from the brain of terminally infected CD1 mice. Limited proteolysis of prion-infected brain homogenate with proteinase K (PK) generates N-terminally truncated PrP^Sc^ 1 (Fig. 1a) while concomitantly destroying PrP^C^ and most other brain proteins. However, although PK has been widely used in prion purification^15^,^16^, not all prion strains show the same degree of PK-resistance and protease-sensitive prions are now well documented^6^,^20^,^22^,^23^. Recently we showed that a different protease (pronase E), when combined with selective protein precipitation using sodium phosphotungstic acid (NaPTA), could generate enriched prion preparations devoid of PrP^C^ that contain PrP^Sc^ in full length form^21^.

**Figure 2:**
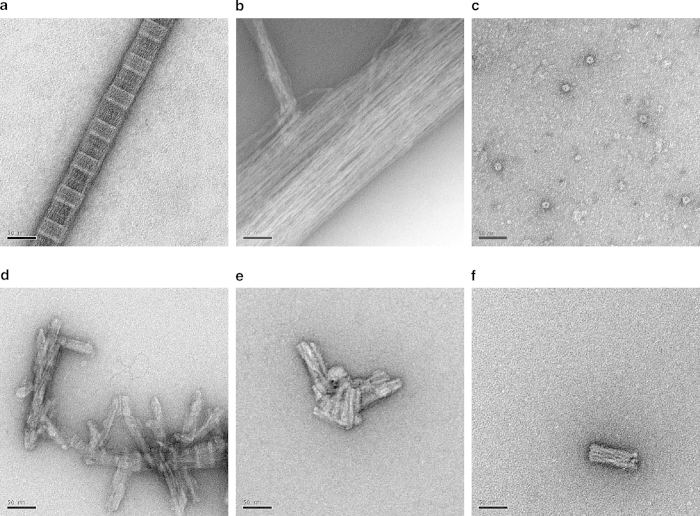
Electron microscopy images of protein structures in purified fractions from RML-infected CD1 mouse brain. (**a–d**) Commonly observed structures in semi-purified fractions, (**a**) collagen, (**b**) intermediate filaments (**c**) ferritin, (**d**) prion rods seen in association with collagen. (**e**,**f**) Purified prion rods (devoid of contaminating protein structures) present in P4 fractions obtained without PK digestion. Samples were stained with uranyl acetate. Scale bar, 50 nm.

As a starting point for method development, we used PK or pronase E digestion, NaPTA precipitation and salt (KI) extraction to generate crude insoluble prion preparations. Typically these contained ~10-20% of the initial RML prion infectivity and were enriched for pathological PrP together with reproducible patterns of contaminating proteins (Figure S1). Excision of these additional proteins from SDS-PAGE gels and their analysis by either mass spectrometry or amino acid sequencing (Figure S1) or inspection of the purified material by electron microscopy (EM) (Fig. 2) identified myelin-associated oligodendrocyte basic protein (MOBP), intermediate filaments (dominated by cytokeratins), collagen and ferritin as the most prominent and persistent contaminants. All these proteins have been previously identified (or observed) as contaminants of murine prions by other researchers^24^,^25^,^26^. Whilst complex separations on density gradients using ultra-centrifugation increase the purity of rodent prions^15^,^16^,^25^,^26^, we found that once MOBP, collagen fibres and intermediate filaments had been co-sedimented with RML prions they could not be differentially extracted or degraded without destroying a significant proportion of the infectious prion titre. Consequently, we sought to remove these proteins from brain homogenate before the first sedimentation of RML prions into a pellet fraction. Surprisingly, by targeting the elimination of MOBP, collagen fibres and intermediate filaments we discovered that highly purified prions could be isolated using simple procedures.

A flow chart of the optimised purification procedure is shown in Fig. 1b. Ten percent (w/v) brain homogenate prepared in phosphate buffered saline was treated with pronase E to digest the majority of PrP^C^ and other brain proteins. The digested sample was then treated with a detergent (sarkosyl) to solubilise protein and an endonuclease (benzonase) to degrade nucleic acid and reduce sample viscosity. Following the addition of NaPTA to increase the density of detergent-insoluble PrP aggregates^6^,^27^ the density medium iodixanol (Optiprep) was added to produce a final concentration of 35% (w/v) in the sample. After centrifugation at 16,100 *g* this sample separated into an insoluble pellet fraction (P1), a clarified supernatant (SN1) and a buoyant, partially flocculated, surface layer (SL) that was highly enriched in lipid and myelin proteins (including MOBP) (Fig. 1b). SN1 had a highly heterogeneous protein composition (Fig. 1c) but contained ~80% of the starting prion infectivity (**Table S1**). The remainder of the prion infectivity was distributed in P1 and SL at a ratio of ~3:1, respectively. Following its careful isolation to avoid cross-contamination with SL or P1, we initially diluted SN1 two-fold with aqueous buffer (to reduce iodixanol concentration to 17.5% (w/v) and tested whether centrifugation at 16,100 *g* could now efficiently sediment prion infectivity. With respect to total protein the resultant pellet was highly enriched for both prion infectivity and pathological PrP, but when visualised by EM, PrP assemblies in these samples (akin to prion rods described by Prusiner and colleagues^1^,^14^) were seen to be contaminated with collagen fibres and intermediate filaments and by ferritin particles (Fig. 2a-d). Based upon the large dimensions of the collagen fibres and intermediate filament bundles relative to the prion rods (Fig. 2) we sought to remove them from SN1 before dilution and centrifugation by simple passage through a 0.45 μm pore sized membrane filter (Fig. 1b). Following this procedure we found that filtered-SN1 contained 50-60% of the starting prion infectivity present in brain homogenate (Table S1) and was devoid of contaminating collagen fibres and intermediate filaments. Subsequently, we diluted the filtered-SN1 sample two-fold (to give 17.5% (w/v) iodixanol) and centrifuged at 16,100 *g*. The resultant, barely visible, pellet (P2) (Fig. 1b) contained about 25% of the starting prion infectivity present in brain homogenate and was highly enriched for pathological PrP (Fig. 1c). EM analyses revealed that prion rods and ferritin particles were the only visible protein structures. Remarkably, the isolation of highly purified prions, devoid of ferritin particles and all other contaminating proteins, was achieved simply by washing the P2 pellet twice in buffer containing 17.5% (w/v) iodixanol to generate a final pellet, P4 (Figs. 1b,c, 2e,f).

**Figure 3:**
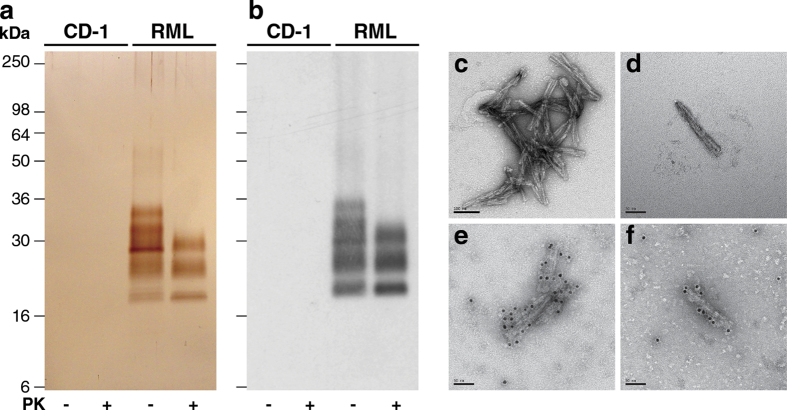
Purified RML prions from CD1 mouse brain. (**a,b**) P4 fractions from uninfected (CD1) or RML-infected (RML) mouse brain purified with (+) or without (–) PK-digestion. (**a**) Silver-stained 16% SDS-PAGE gel. (**b**) Western blot using anti-PrP monoclonal antibody ICSM35. The equivalent of 200 μl (**a**) or 20 μl (**b**) of 10% (w/v) brain homogenate was loaded per lane. (**c-f**) Electron microscopy images of prion rods in P4 fractions from RML-infected CD1 mouse brain obtained without PK digestion. Samples were stained with uranyl acetate. Prion rods in panels **e** and **f** have been labelled with anti-PrP monoclonal antibody SAF-32 and an anti-mouse IgG secondary antibody conjugated to 10 nm gold particles. Scale bar, (**c**) 100 nm, (**d–f**) 50 nm.

P4 fractions from RML-infected CD1 mouse brain contained pathological PrP at >99% purity with respect to total protein (Figs. 1c, 3a) with all major SDS-PAGE silver-stained bands immuno-reactive with an anti-PrP monoclonal antibody on western blot (Fig. 3b). EM analysis of P4 samples revealed that prion rods were the only visible protein structures (Fig. 3c,d) and that these could be specifically gold-labelled using an anti-PrP monoclonal antibody (Fig. 3e,f). In sharp contrast, P4 samples from normal, uninfected CD1 mouse brain showed no detectable protein (Fig. 3a) or visible protein structures by EM (data not shown). P4 contained a yield of 12 ± 0.3% of the total PrP present in starting RML brain homogenate and a yield of 10 ± 1.5% of the starting infectious prion titre (measured by SCA in Pk1/11 cells; mean ± SEM; n = three preparations). End-point titration of the P4 fraction in transgenic Tg20 mice (that over-express mouse PrP^28^) reported RML prion titres that corresponded closely with those determined in cell culture and a specific prion infectivity of 10^8.8 ± 0.2^ intra-cerebral LD_50_/mg protein (Figure S2). Digestion of P2 with PK followed by washing (Fig. 1b) to produce a PK-digested P4 pellet showed that the purified PrP was highly protease-resistant (Figs. 1c, 3a,b); >90**%** the total PrP and the prion infectivity was preserved after this treatment. Because ~15-20% of the total PrP present in starting RML brain homogenate is PK-resistant^20^,^21^ the method described here provides a yield of >50% of this pathological PrP fraction. To test general applicability, we then examined the ability of the method to purify prions from the brain of different hosts propagating other prion strains.

### Purification of prions from hamster and human brain

**Figure 4:**
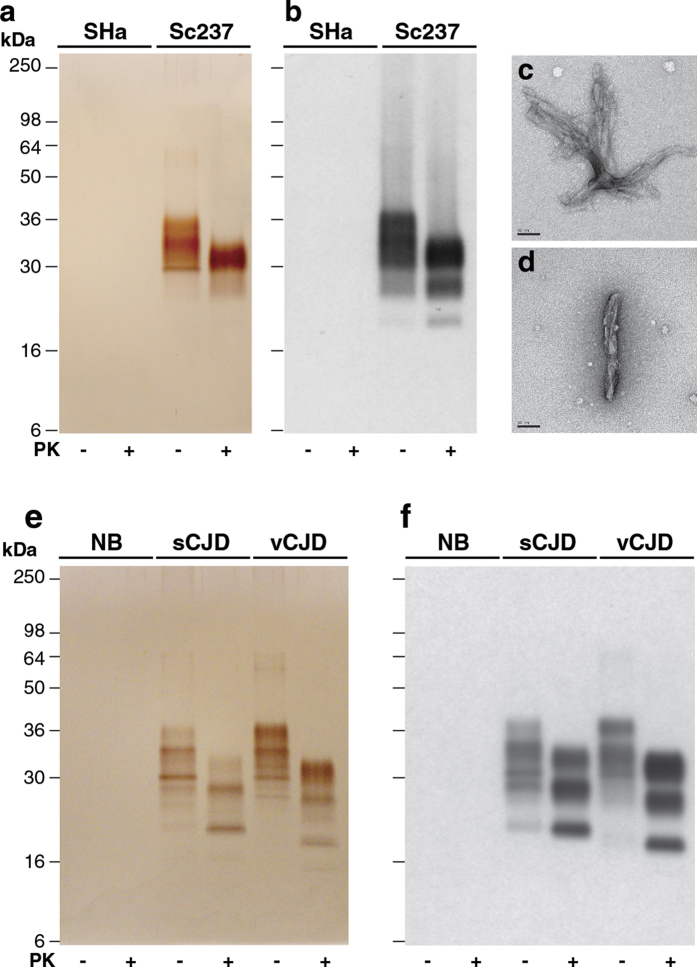
Purified prions from hamster or human brain. (**a**,**b**) P4 fractions from uninfected (SHa) or Sc237 prion-infected (Sc237) Syrian hamster brain purified with (+) or without (–) PK-digestion. (**a**) Silver-stained 16% SDS-PAGE gel. (**b**) Western blot using anti-PrP monoclonal antibody ICSM35. The equivalent of 200 μl (**a**) or 20 μl (**b**) of 10% (w/v) brain homogenate was loaded per lane. (**c**,**d**) Electron microscopy images of prion rods in the P4 fraction from Sc237 prion-infected Syrian hamster brain obtained without PK digestion. Scale bar, 50 nm. (**e**,**f**) P4 fractions from the frontal cortex of normal human brain (NB), sporadic CJD brain (sCJD) or variant CJD brain (vCJD). (**e**) Silver-stained 16% SDS-PAGE gel. (**f**) Western blot using anti-PrP monoclonal antibody 3F4. The equivalent of 200 μl (**e**) or 20 μl (**f**) of 10% (w/v) brain homogenate was loaded per lane.

We used the optimised protocol (Fig. 1b) to purify Sc237 prions from the brain of terminally infected Syrian hamsters. Resultant P4 samples contained highly purified, predominantly full length, pathological hamster PrP (Fig. 4a,b) with a recovery of 14.2 ± 1.2% (mean ± SEM; n = three preparations) of the total PrP present in starting Sc237 brain homogenate. The majority of this purified PrP (~90%) was resistant to PK digestion (Fig. 4a,b) and this treatment generated the typical pattern of N-terminally truncated PrP^Sc^ glycoforms that characterises the Sc237 prion strain (Fig. 4a,b). EM analysis revealed that prion rods were the only visible protein structures (Fig. 4c,d). P4 samples from normal, uninfected hamster brain showed no detectable protein (Fig. 4a,b) or visible protein structures by EM (data not shown). As cell based bioassay of Sc237 prions has not yet been established we end-point titrated P4 fractions in Syrian hamsters (Figure S3). Purified Sc237 prions associated with full-length pathological PrP had a specific prion infectivity of 10^10.5 ± 0.3^ intra-cerebral LD_50_/mg protein which agrees closely with previously published findings^29^.

Next we applied the purification protocol to human brain. We generated P4 samples from the frontal cortex of patients with neuropathologically confirmed sporadic CJD (*PRNP* codon 129 methionine homozygous with type 2 PrP^Sc^ by the London classification^30^) or variant CJD. P4 samples contained highly purified pathological PrP, predominantly in full length form, with all SDS-PAGE silver-stained bands immuno-reactive with an anti-PrP monoclonal antibody on western blot (Fig. 4e,f). In contrast, P4 samples from normal human frontal cortex contained no detectable protein (Fig. 4e,f). Generation of PK-digested P4 samples (Fig. 4e,f) showed the expected differences in N-terminally truncated PrP^Sc^ fragment sizes and glycoform ratios that distinguish these distinct PrP^Sc^ molecular strain types^30^. Unfortunately, due to biohazard restrictions, EM imaging of these samples has not yet been possible. Bioassay and strain typing studies of purified human prions in transgenic mice overexpressing human PrP^31^ is on-going and will be reported elsewhere.

### Purified RML and ME7 prions retain their prion strain properties

**Figure 5:**
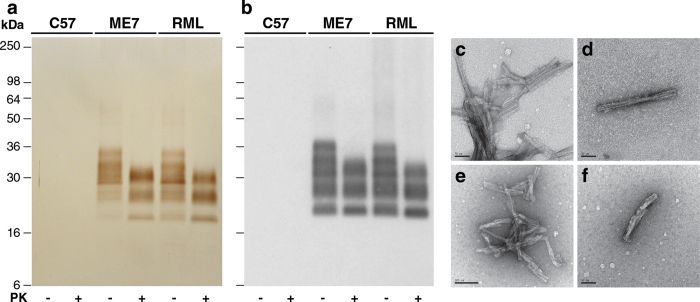
Purified ME7 and RML prions from C57Bl/6 mouse brain. (**a,b**) P4 fractions from uninfected (C57), ME7 prion-infected (ME7) or RML prion-infected (RML) C57Bl/6 mouse brain purified with (+) or without (–) PK-digestion. (**a**) Silver-stained 16% SDS-PAGE gel. (**b**) Western blot using anti-PrP monoclonal antibody ICSM35. The equivalent of 200 μl (**a**) or 20 μl (**b**) of 10% (w/v) brain homogenate was loaded per lane. (**c**-**f**) Electron microscopy images of prion rods in P4 fractions obtained without PK digestion; (**c**,**d**) ME7 prions, (**e**,**f**) RML prions. Scale bar, (**c**,**d**,**f**) 50 nm, (**e**) 100 nm.

Having established the ability of the purification protocol to isolate different prion strains from different hosts we sought to examine the strain properties of purified prions and thereby validate the utility of the method. We propagated the RML and ME7 prion strains in C57Bl/6 mice (Figure S4) and purified prions from the brain of terminally infected mice using the optimised protocol (Fig. 1b). P4 samples contained highly purified pathological PrP (Fig. 5a,b) with recoveries of 9.7 ± 0.5% and 13.6 ± 0.7% (mean ± SEM; n = six preparations) of the total PrP present in the starting RML or ME7 brain homogenate, respectively, which corresponded with yields of 9 ± 1% and 17 ± 1% (mean ± SEM; n = six preparations) of the prion infectivity present in starting RML or ME7 brain homogenate, respectively. EM analysis revealed prion rods as the only visible protein structures (Fig. 5c-f) whereas P4 samples from normal, uninfected C57Bl/6 mouse brain showed no detectable protein (Fig. 5a,b) or visible protein structures by EM (data not shown). Generation of PK-digested P4 samples (Fig. 5a,b) showed the formation of N-terminally truncated PrP^Sc^ possessing the characteristic PrP glycoform ratios that distinguish the RML and ME7 prion strains (Figures S4 and S5).

**Figure 6:**
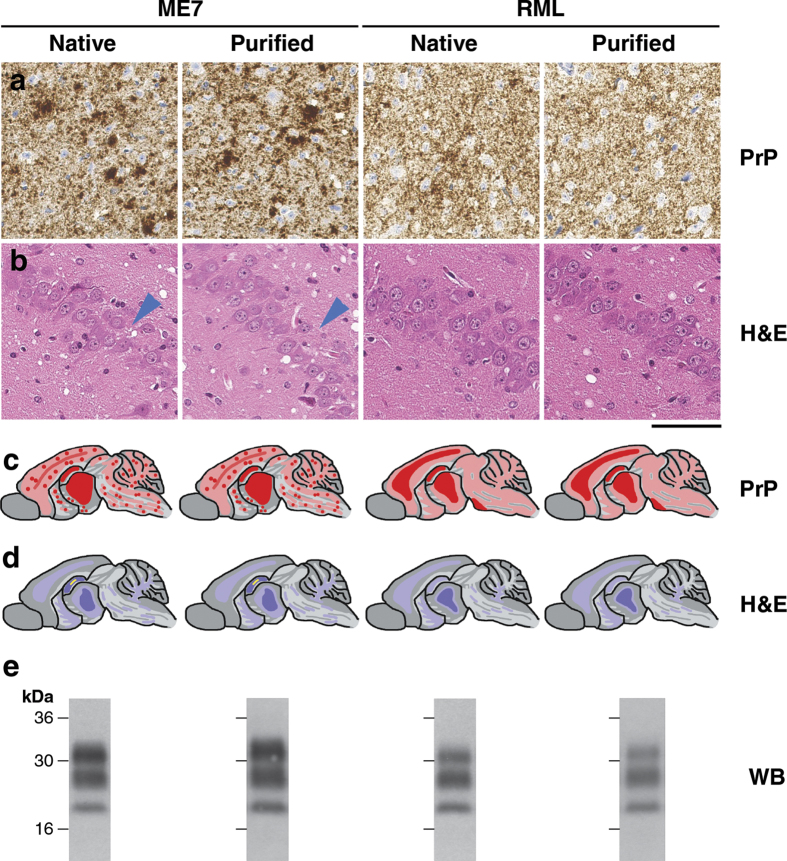
Transmission of native or purified ME7 and RML prions to C57Bl/6 mice. ME7 or RML prions in native form (from 10% (w/v) brain homogenate) or purified form (from P4 fractions obtained without PK-digestion) (Fig. 5 and S5) were inoculated intracerebrally into C57Bl/6 mice. Brains from five mice with end-stage clinical prion disease from each transmission group were analysed to compare neuropathological changes, the patterns of abnormal PrP deposition and the molecular strain types of PrP^Sc^ that propagated in the disease. Data presented are representative of those found in all brains examined from each transmission group. (**a**) Immunohistochemistry showing abnormal prion protein (PrP) deposition in the cortex using anti-PrP monoclonal antibody ICSM35. Scale bar, 50 μm. (**b**) Haematoxylin and eosin (H&E) staining of the hippocampus showing spongiform change associated with both ME7 and RML prion infection and neuronal drop-out associated with ME7 prions (blue arrowheads). Scale bar, 50 μm. (**c**,**d**) Schematic representations of mouse brain. (**c**) Abnormal PrP deposition; pink shading, moderate PrP deposition, red shading, intense PrP deposition, red dots, PrP microplaques. (**d**) Spongiform change; light blue shading, widely dispersed spongiosis, blue shading, intense focal spongiosis, yellow shading, neuronal loss. (**e**) Representative western blots of PK-digested 10% (w/v) brain homogenate using anti-PrP monoclonal antibody ICSM35. A predominance of di-glycosylated PrP or mono-glycosylated PrP is associated with ME7 or RML prions, respectively. Quantitation of PrP glycoforms is provided in Figure S6.

To examine the prion strain properties of the purified RML and ME7 prions we inoculated these samples or native prions from the original starting RML and ME7 brain homogenates into further groups of C57Bl/6 mice (Figure S5). Attack rates and mean survival times are reported in Figure S5 and for each prion strain the native or purified prions showed no significant difference in their transmission properties. Comparative neuropathological and biochemical analyses of terminal mouse brain from these transmissions (Fig. 6 and S6) indicated that the strain properties of ME7 and RML prions were essentially unaltered by purification. For the ME7 prion strain, the defining characteristics in terminal C57Bl/6 mouse brain are the deposition of disease-related PrP both diffusely and as micro-plaques throughout the brain, a loss of neurons in the hippocampus and the presence of PK-resistant PrP^Sc^ showing a predominance of di-glycosylated PrP. In contrast, the defining characteristics of the RML prion strain in terminal C57Bl/6 mouse brain are predominantly diffuse deposition of disease-related PrP throughout the brain (with little or no PrP microplaques), minimal loss of neurons in the hippocampus and the presence of PK-resistant PrP^Sc^ showing a predominance of mono-glycosylated PrP. Because these defining strain characteristics were equivalently observed following transmission of native or purified RML and ME7 prions (Fig. 6 and S6) we conclude that the purification method reported here does not cause any significant alteration of prion composition or structure.

## Discussion

Despite enormous advances in our understanding of prion biology the precise composition and structure of a mammalian prion remain unknown. A major obstacle to achieving this goal has been the inability to readily isolate intact prions from brain for structural study. The method reported here now facilitates the rapid isolation of prions to levels of purity comparable with, or exceeding, those generated using the best historical protocols. The lack of non-PrP proteins in purified prion samples which maintain their prion strain transmission properties is consistent with strains being encoded by distinct PrP conformations and assembly states^1^,^2^,^3^,^4^,^5^,^6^ and supports recent mass spectrometry data suggesting that no strain-specific protein co-factors are associated with disease-related PrP^32^,^33^. Importantly, the technical simplicity of the method, coupled with the requirement for only small quantities of prion-infected brain tissue, suggests that the method will be generally practicable and will facilitate structural studies of prions that have previously been unfeasible. Elucidation of the structure of infectious mammalian prions and the determinants of strain will provide a major advance to understanding the molecular mechanism of prion replication and will have direct translational benefits for both rational therapeutics and prion disease diagnosis. Defining key structural differences between infectious and non-infectious PrP fibrils^34^ will also be of great interest with respect to a wide range of other neurodegenerative diseases involving protein misfolding in which there is also growing evidence for strains^35^,^36^,^37^,^38^,^39^,^40^,^41^.

## Methods

### Research governance

Work with prion-infected samples was conducted in microbiological containment level 3 facilities with strict adherence to safety protocols. Work with animals was performed in accordance with licences approved and granted by the UK Home Office (Project Licences 70/6454 and 70/7274) and conformed to University College London institutional and ARRIVE guidelines. Storage and biochemical analysis of human tissue samples and transmission studies to mice were performed with informed consent from all patients or relatives. All experimental protocols were approved by the Local Research Ethics Committee of UCL Institute of Neurology/National Hospital for Neurology and Neurosurgery and complied with the code of practice specified in the Human Tissue Authority licence held by UCL Institute of Neurology.

### Reagents

Dulbecco’s phosphate buffered saline lacking Ca^2+^ or Mg^2+^ ions (D-PBS) was supplied by Invitrogen (Prod. No. 14190-086). All solutions were filtered using a Stericup-GP filter unit (0.22 μm pore size polyether sulfone membrane; Millipore) and stored at room temperature. Protease type XIV (pronase E) from Streptomyces griseus was obtained as a lyophilized powder from Sigma-Aldrich (Prod. No. P5147). The specific enzymatic activity is approximately 4 units/mg (where 1 unit liberates 1.0 mmol of tyrosine/min at pH 7.5 at 37 °C using casein as a substrate). Proteinase K (EC 3.4.21.64) from Tritirachium album limber was obtained freeze-dried from Merck (Prod. No. 70663). The specific enzymatic activity is approx 30 Anson units/g (where 1 Anson unit is the amount of enzyme that liberates 1 mmol of Folin-positive amino acids/min at pH 7.5 and 35 °C, using haemoglobin as a substrate). Pronase E and proteinase K stocks were prepared in water at concentrations of 10 and 0.1 mg/ml, respectively and aliquots stored at −70 °C. Sodium lauroylsarcosine (sarkosyl) was supplied by Calbiochem. Benzonase Nuclease (purity > 99%; 25 U/ml) was supplied by Novagen. Optiprep (60% (w/v) iodixanol in water) and sodium phosphotungstic acid (NaPTA) were obtained from Sigma-Aldrich.

### Prion sources

Three batches of 200 brains from CD-1 mice terminally-affected with the RML prion strain were homogenized in D-PBS using tissue grinders to produce three ~1 L pools of 10% (w/v) RML brain homogenate (designated I6200, I8700 and I13100). All three pools showed comparable prion infectivity titres in the Scrapie Cell End Point Assay (SCEPA) (approximately 10^6.5^ tissue culture infectious units (TCIU) / ml in PK1/11 cells^20^,^21^ or 10^7.7^ TCIU / ml in PK1/2 cells^19^. I6200 reported a prion titre of 10^7.3 ± 0.5^ (mean ± SD) intracerebral LD_50_ units / ml when endpoint titrated six times in Tg20 mice and I8700 reported a prion titre of 10^7.2^ intracerebral LD_50_ units / ml when endpoint titrated once in Tg20 mice. Four batches of ~90 ml of 10% (w/v) normal CD-1 mouse brain homogenate prepared in D-PBS (designated I7219, I8402, I10340 or I14040) were used as controls and showed no detectable prion infectivity in either the SCA or Tg20 bioassay. Brains from 75 Syrian hamsters terminally-infected with Sc237 prions were used to generate a ~750 ml pool of 10% (w/v) Sc237 brain homogenate in D-PBS (designated I9200) that reported a prion titre of 10^8.2^ intracerebral LD_50_ units / ml when endpoint titrated once in Syrian hamsters. One pool of ~50 ml of 10% (w/v) normal Syrian hamster brain homogenate in D-PBS (designated I10500) and a second 10% (w/v) homogenate prepared from a single normal Syrian hamster brain (designated I16169) were used as controls and showed no detectable prion infectivity in Syrian hamster bioassay. ME7 and RML prion strains were propagated in C57Bl/6 mice (C57BL/6JOlaHsd; Harlan, UK). 30 brains from terminally affected mice were used to generate pools of ~150 ml 10% (w/v) RML or ME7 brain homogenate prepared in D-PBS (designated I14050 ME7 and I14051 RML) that both reported approximate titres of 10^5.0^ tissue culture infectious units / ml in the SCEPA using LD9 cells. One pool of ~150 ml 10% (w/v) normal C57BL/6 mouse brain homogenate (designated I14052) was used as a control and showed no detectable prion infectivity in either the SCEPA or C57BL/6 mouse bioassay. Brain from patients with neuropathologically confirmed variant CJD or sporadic CJD or from individuals without neurological disease were used to generate 10% (w/v) frontal cortex (grey matter) homogenates prepared in D-PBS. Brain homogenates were stored as aliquots at −70 °C.

### Prion purification

Incubations were performed in an Eppendorf Thermomixer Comfort (1.5 ml tube block) with constant agitation at 800 rpm. Centrifugation was performed at 37 °C in an Eppendorf 5424R microfuge. 200 μl aliquots of 10% (w/v) brain homogenate were dispensed into standard 1.5 ml microfuge tubes with screw cap and rubber O ring. Typically, 12 tubes were processed at a time. Samples were treated with 2 μl of 10 mg/ml pronase E prepared in water (to give 100 μg/ml final protease in the sample) and incubated for 30 min at 37 °C. Samples were then treated with 4.1 μl of 0.5 M EDTA prepared in water pH 8.0 to give 10 mM final concentration in the sample. 206 μl of 4% (w/v) sarkosyl in D-PBS and 0.83 μl of Benzonase were then added to give final concentrations in the sample of 2% (w/v) and 50 U/ml, respectively. Following incubation for 10 min at 37 °C, 33.5 μl of 4% (w/v) NaPTA prepared in water pH 7.4 was added to give a final concentration of 0.3% (w/v) in the sample. After incubation for 30 min at 37 °C the samples were adjusted (and thoroughly mixed) with 705.3 μl of 60% (w/v) iodixanol and 57. 2 μl of 4% (w/v) NaPTA prepared in water pH 7.4 to give final concentrations in the sample of 35% (w/v) and 0.3% (w/v), respectively. After centrifugation for 90 minutes at 16,100 *g* the sample separates into an insoluble pellet fraction (P1), a clarified supernatant (SN1) and a buoyant, partially flocculated, surface layer (SL). 1 ml of SN1 was carefully isolated from each tube taking extreme care to avoid cross contamination with either P1 or SL. SN1 was filtered using an Ultrafree-HV microcentrifuge filtration unit (0.45 μm pore size Durapore membrane, Millipore, Prod. No. UFC30HV00). This was accomplished by loading 500 μl aliquots of SN1 and centrifugation at 12,000 *g* for 30 sec using one filtration unit per ml of SN1. 480 μl aliquots of filtered SN1 were transferred to new 1.5 ml microfuge tubes and thoroughly mixed with an equal volume of 2% (w/v) sarkosyl in D-PBS containing 0.3% (w/v) NaPTA pH 7.4 and incubated for 10 min at 37 °C. Samples were then centrifuged for 90 min at 16,100 *g* to generate an insoluble pellet fraction (P2) and a clarified supernatant (SN2). SN2 was carefully removed and discarded, after which each P2 pellet was resuspended in 10 μl of wash buffer composed of D-PBS, containing 17.5% (w/v) iodixanol and 0.1% (w/v) sarkosyl. Resuspended P2 pellets were pooled and dispensed as 20 μl aliquots. At this point samples may be frozen at −70 °C. These samples contain partially purified prions in approximately one tenth of the volume of the starting 10% (w/v) brain homogenate from which they were derived. Final washing of P2 samples was performed as follows. 20 μl aliquots of the pooled P2 pellet were thoroughly mixed with 180 μl of wash buffer (see above) followed by the addition of 16.2 μl of 4% (w/v) NaPTA (prepared in H_2_O; pH 7.4) to give 0.3% (w/v) final concentration in the sample. Samples were centrifuged at 16,100 *g* for 30 min to generate a clarified supernatant (SN3) and a barely visible pellet fraction (P3). SN3 was carefully removed and discarded after which P3 was resuspended in 200 μl of wash buffer followed by the addition of 16.2 μl of 4% (w/v) NaPTA (prepared in H_2_O; pH 7.4) to give 0.3% (w/v) final concentration in the sample. Samples were centrifuged at 16,100 *g* for 30 min to generate a clarified supernatant (SN4) and a barely visible pellet fraction (P4). SN4 was carefully removed and discarded. Each P4 pellet was resuspended in 20 μl of D-PBS containing 0.1% (w/v) sarkosyl after which they were pooled, dispensed as 20 μl aliquots and stored at −70 °C. These samples contain purified prions in approximately one tenth of the volume of 10% (w/v) brain homogenate from which they were derived. As the method produces a recovery of ~10% of both the starting prion titre and PrP content these samples have a PrP concentration and prion titre closely similar to the starting 10% (w/v) brain homogenate.

PK digestion is not an obligatory step in the method and is optionally performed on the P2 fraction. 20 μl of pooled P2 sample (see above) was treated with 2.2 μl of 0.1 mg/ml PK prepared in water (to give 10 μg/ml final protease concentration in the sample) and incubated at 37 °C for 1 h. Digestion was terminated by addition of 0.22 μl of 100 mM 4-(2-Aminoethyl) benzenesulfonyl fluoride hydrochloride (AEBSF) to give 1 mM final concentration in the sample, followed by washing as described above to generate PK-digested P4 pellet fractions.

### SDS-PAGE, immunoblotting and silver stain

Samples were mixed with an equal volume of 2 x SDS sample buffer (125 mM Tris-HCl, 20% (v/v) glycerol pH 6.8 containing 4% (w/v) SDS, 4% (v/v) 2-mercaptoethanol, 8 mM AEBSF and 0.02% (w/v) bromophenol blue) and immediate transfer to a 100 °C heating block for 10 min. Electrophoresis was performed on 16% (w/v) Tris-glycine gels (Invitrogen), run for 80 min at 200V, prior to electroblotting to Immobilon P membrane (Millipore) (90 min at 35 V or 16 h at 15 V). Membranes were blocked in 1 x phosphate buffered saline (PBS) (prepared from 10 x concentrate, VWR International, Prod No.43711 7K) containing 0.05% (v/v) Tween 20 (PBST) and 5% (w/v) non-fat dried skimmed milk powder and then probed with 0.2 μg/ml anti-PrP monoclonal antibody in PBST for at least 1 h. ICSM35 (D-Gen Ltd) was used for rodent PrP and 3F4 (Cambridge Bioscience) for human PrP. After washing (1 h with PBST) the membranes were probed with a 1:10,000 dilution of alkaline-phosphatase-conjugated goat anti-mouse IgG secondary antibody (Sigma-Aldrich Prod. No. A2179) in PBST. After washing (1 h with PBST and 2 × 5 min with 20 mM Tris pH 9.8 containing 1 mM MgCl_2_) blots were incubated for 5 min in chemiluminescent substrate (CDP-Star; Tropix Inc) and visualized on Biomax MR film (Kodak). For analysis of PrP glycoforms, blots were developed in chemifluorescent substrate (AttoPhos; Promega) and visualised on a Storm 840 phosphoimager (Amersham) using ImageQuaNT software (Amersham). For purified prion preparations, typical loading was the equivalent of 20 μl of 10% (w/v) brain homogenate per lane. SDS-PAGE gels (prepared as above) were silver stained using the PlusOne Protein Silver Stain Kit (GE Healthcare) according to the manufacturer’s instructions. Gels were photographed on a light box using a Nikon Coolpix P6000 digital camera. For purified prion preparations, typical loading was the equivalent of 200 μl of 10% (w/v) brain homogenate per lane.

### ELISA

PrP content in brain homogenate and purified samples was determined by enzyme-linked immunosorbent assay (ELISA). Samples were denatured by mixing with an equal volume of 4% (w/v) SDS prepared in water and heating at 100 °C for 10 min. After cooling, 20 μl of the denatured sample was mixed thoroughly with 600 μl of an ELISA capture buffer (50 mM Tris/HCl; pH 8.4 containing 2% (w/v) bovine serum albumin (Fraction V, protease free, Sigma–Aldrich), 2% (v/v) Triton X-100 and 2% (w/v) sarkosyl). Further dilutions of the sample (see below for linear working range of the assay) were performed in capture buffer.

Black 96-well ELISA plates (Greiner Bio-One) were coated for 16 h at 4 °C with 100 μl/well of carbonate buffer (35 mM sodium bicarbonate, 15 mM sodium carbonate in water; pH 9.6) containing 0.2 μg/ml anti-PrP monoclonal antibody ICSM18 (D-Gen Ltd). The wells were then washed three times with PBST using an automated microplate washer (Tecan) and blocked by the addition of 300 μl/well of Superblock T20 (Pierce Protein Research Products) and incubated for 1 h at 37 °C with constant agitation. The plate was washed once with 300 μl of PBST before denatured samples (in ELISA capture buffer; see above) were transferred to triplicate wells (50 μl sample/well) and incubated for 1 h at 37 °C with constant agitation. The plate was then probed with PBS containing 1% (v/v) Tween 20 and 1 μg/ml biotinylated anti-PrP monoclonal antibody ICSM 35 (100 μl/well) and incubation at 37 °C for 1 h with constant agitation. Wells were washed a further three times with 300 μl of PBST, followed by the addition of 100 μl of PBS containing 1% (v/v) Tween 20 and a 1:10,000 dilution of High Sensitivity NeutrAvidin HRP Conjugate (Thermo Scientific Prod No. 31030) and incubation for 30 min at 37 °C. Wells were washed four times with 300 μl of PBST and developed with 100 μl/well of QuantaBlu working solution (Pierce Protein Research Products) for 1 h after which the reaction was stopped by the addition of 100 μl/well of QuantaBlu stop solution (Pierce). Fluorescence (λex = 340 nm, λem = 420 nm) was measured on a Tecan Ultra Evolution microplate reader. Brain homogenate samples were background corrected by subtraction of the signal produced from processing an equivalent aliquot of brain homogenate prepared from Prnp^o/o^ mice. All other samples were corrected by the subtraction of an appropriate buffer control. PrP content for all samples was quantified by correlation with a serial dilution curve of recombinant mouse PrP amino acid residues 23-231. We used a working detection range in the assay of typically 0.5 to 2.5 ng of recombinant PrP per well. 10% (w/v) RML infected brain homogenate contains ~5.5 μg/ml total PrP so that a loading per well of 0.2 μl of denatured 10% (w/v) RML brain homogenate (in 50 μl samples of ELISA capture buffer) falls in the middle range of the assay. Purified prion preparations were resuspended in volumes appropriate to this concentration range based upon 10% recovery of PrP from 10% (w/v) brain homogenate.

### Electron microscopy

300 mesh carbon coated copper grids (Electron Microscopy Sciences) were glow discharged for 40 sec using an EMS 100 x glow discharge unit (Electron Microscopy Sciences, USA) and then loaded with prion-infective material within class 1 microbiological safety cabinets situated in microbiological containment level 3 facilities. Purified prion preparations (and controls) were resuspended in 20 mM Tris, 150 mM NaCl pH 7.4 (TBS) typically at 1/25 of the volume of 10% brain homogenate from which they were derived. Buffer exchange was routinely accomplished by centrifuging P4 samples at 16,100 *g* for 30 min discarding the supernatant and then resuspending the pellet fraction in the desired volume of buffer. Two minutes after loading the sample, grids were blotted with filter paper (Whatman, Grade 1), washed briefly in two drops of water and then stained with 2% (w/v) uranyl acetate prepared in water (Agar Scientific) for 45 sec. Excess stain was removed with filter paper and grids allowed to dry in air. Dried grids were sealed into approved packaging (UN 3373 Biological Substance, Category B) for transport between laboratories. Grids containing immobilized prions were inserted into the microscope using a dedicated sample holder with strict adherence to local risk assessment and safety protocols. Samples were examined on a Tecnai 10 Transmission Electron Microscope (FEI, Eindhoven, NL) at an accelerating voltage of 100 kV. Digital images were typically recorded using a 1K Gatan Multiscan 794 CCD camera at a nominal magnification of 44,000 X with a typical defocus of 300 nm and analyzed using Gatan Digital Micrograph (Gatan Inc).

Immunolabelling of prion rods was performed as follows. Purified prions isolated from 0.5 ml 10% (w/v) brain homogenate were resuspended in 500 μl TBS containing 0.1% (w/v) sarkosyl and 1 μg/ml anti-PrP monoclonal antibody SAF-32 (Cayman Chemical Company) and incubated for 16 h at 25 °C with gentle agitation. 40.5 μl of 4% (w/v) NaPTA (prepared in H_2_O; pH 7.4) was then added to give 0.3% (w/v) final concentration in the sample. The sample was then centrifuged at 16,100 *g* for 30 min and the supernatant discarded after which the pellet was resuspended in 9.5 μl of TBS containing 5% (v/v) glycerol and 0.5 μl of goat anti-mouse IgG conjugated to 10 nm gold particles (Sigma; Prod No. G7652) was then added and incubated at 25  °C for 3 hours with gentle agitation. The sample was pulse centrifuged for 5 sec then loaded onto glow-discharged carbon coated grids and stained with uranyl acetate as described above. Specificity of the labelling of prion rods by the SAF-32 anti-PrP monoclonal antibody was established by demonstrating that labelling was abolished when the procedure was changed in the following ways. (1) Inclusion of a 100-fold molar excess of recombinant mouse PrP (amino acid residues 23-231) during SAF-32 incubation. (2) Replacement of SAF-32 with an anti-keratin monoclonal antibody (Cell Signalling Technology Prod. No. 4545). (3) Omission of SAF-32 and incubation with goat anti-mouse IgG-gold conjugate only.

### Scrapie cell assay

Prion infectivity in RML and ME7 preparations was quantified using the scrapie cell assay with PK1/11 cells (for RML prions) or LD9 cells (for ME7 prions)^17^,^42^. Samples were prepared at appropriate dilutions in OptiMEM (Invitrogen) for PK1 cells and MEM (Sigma-Aldrich) for LD9 cells. Cells were exposed to the samples for 3 days before being split 1:8 into fresh cell culture media and grown back to confluence. Two further splits were conducted before transferring a sample of the cells to ELISPOT plates for analysis of the number of cells containing PK-resistant PrP. Infectivity of purified prion samples was calculated by correlation with the serial dilution of 10% (w/v) brain homogenate from which they were derived. We use a working detection range in the assay of typically 50 to 1,000 intracerebral LD_50_ units per well.

### Rodent bioassay

CD1 mice, C57Bl/6 mice and Syrian hamsters were supplied by Harlan, UK. Tg20 transgenic mice^28^ (overexpressing mouse PrP on a *Prnp*^*o/o*^ background) were from in-house breeding stock. 10% (w/v) brain homogenate was diluted to 1% (w/v) in D-PBS and thereafter serially diluted ten-fold using 1% (w/v) normal brain homogenate in D-PBS as diluent. Purified prion samples (of known PrP concentration) in D-PBS containing 0.1% (w/v) sarkosyl were serially diluted 10^-1^ to 10^-8^ using 1% (w/v) normal brain homogenate in D-PBS as diluent. 30 μl aliquots of samples were inoculated intracerebrally into recipient animals as described previously^43^. Animals were examined daily and were killed if exhibiting signs of distress or once a diagnosis of clinical prion disease was established. At post-mortem, brain was removed and divided sagittally, with half frozen at −70 °C and half fixed in 10% buffered formol-saline at room temperature. Infectious prion titres (LD_50_) were calculated from the attack rates for clinical prion disease using the Reed-Müench formula^44^.

### Immunohistochemistry

Brain fixed in 10% buffered formol-saline was immersed in 98% formic acid for 1 h and paraffin wax embedded. Serial sections (4 μm thick) were pre-treated by boiling for 10 min in a low ionic strength buffer (2.1 mM Tris, 1.3 mM EDTA, 1.1 mM sodium citrate, pH 7.8) before exposure to 98% formic acid for 5 min. Abnormal PrP accumulation was examined using anti-PrP monoclonal antibody ICSM 35 (D-Gen Ltd, London) on an automated IHC staining machine (Ventana Medical Systems Inc., Tucson, Arizona) using proprietary secondary detection reagents (Ventana Medical Systems Inc) before development with 3'3 diaminobenzedine tetrachloride as the chromogen. Conventional methods were used for Harris haematoxylin and eosin staining. Appropriate positive and negative controls were used throughout. Photographs were taken on an ImageView digital camera and composed with Adobe Photoshop.

## Author Contributions

J.D.F.W directed the study and designed the experiments together with A.W., C.T. and J.C. A.W., C.T., N.G., S.J., L.D’C., S.C. and J.D.F.W. developed the purification method and did the biochemistry. A.W., N.G. and J.S. performed the cell culture infectivity assays. J.M.L and S.B. performed the neuropathological analyses of brain. C.T. and S.P performed the electron microscopy analyses under the supervision of H.R.S. J.D.F.W. and A.W. drafted the manuscript with contributions from all authors.

## Additional Information

**How to cite this article**: Wenborn, A. *et al.* A novel and rapid method for obtaining high titre intact prion strains from mammalian brain. *Sci. Rep.* **5**, 10062; doi: 10.1038/srep10062 (2015).

## Supplementary Material

Supplementary Information
