# Supplementary material for: A novel and rapid method for obtaining high titre intact prion strains from mammalian brain
Source: Sci Rep. 2015 May 7;5:10062. doi: 10.1038/srep10062 (PMC4423448; doi:10.1038/srep10062)
Supplement: Supplementary Information [file srep10062-s1.pdf]

Supplementary Information

A novel and rapid method for obtaining high titre intact prion strains from mammalian brain

Adam Wenborn[1], Cassandra Terry[1], Nathalie Gros[1,3], Susan Joiner[1], Laura D'Castro[1,4], Silvia Panico[2,5], Jessica Sells[1], Sabrina Cronier[1,6], Jacqueline M Linehan[1], Sebastian Brandner[1], Helen R Saibil[2], John Collinge[1] & Jonathan D F Wadsworth[1*]

[1]MRC Prion Unit and Department of Neurodegenerative Disease, UCL Institute of Neurology, National Hospital for Neurology and Neurosurgery, Queen Square, London WC1N 3BG, UK

[2]Department of Crystallography and Institute of Structural and Molecular Biology, Birkbeck College, University of London, Malet Street, London WC1E 7HX, UK

[3]Present address: Centre d'étude d'agents Pathogènes et Biotechnologies pour la Santé, Montpellier, France

[4]Present address: MIMS (Hong Kong) Ltd, Wanchai, Hong Kong.

[5]Present address: Department of Biochemistry, Imperial College London.

[6]Present address: INRA, Virologie et Immunologie Moléculaires, Jouy-en-Josas, France

[*]Correspondence should be addressed to J.D.F.W. (j.wadsworth@prion.ucl.ac.uk)

List of Supplementary Information

Table S1	RML prion infectivity and PrP content in fractions from the optimised purification method
Figure S1	Identification of proteins that co-purify with mouse RML prions.
Figure S2	End-point titration of purified RML prions in Tg20 mice.
Figure S3	End-point titration of purified hamster Sc237 prions in hamsters.
Figure S4	Propagation of ME7 and RML prion strains in C57Bl/6 mice.
Figure S5	Transmission of native and purified RML and ME7 prions to C57Bl/6 mice.
Figure S6	PrP glycoform ratios in C57Bl/6 mice inoculated with native or purified RML and ME7 prions.

Table S1. RML prion infectivity and PrP content in fractions from the optimised purification method*

Sample	Infectivity[†] (%)	PrP content[†] (%)
RML brain homogenate[‡]	100 ± 4	100 ± 5
Pronase and detergent treated brain homogenate[§]	112 ± 3	90 ± 5
Pellet 1 (P1)	17 ± 1	14 ± 1
Surface layer (SL)	6 ± 0.5	15 ± 1
Supernatant 1 (SN1)	80 ± 1	78 ± 1
Filtered SN1	55 ± 4	63 ± 3
Pellet 2 (P2)	26 ± 2	44 ± 3
Pellet 4 (P4)[‖]	10 ± 1.5	12 ± 0.3

*Please refer to Figure 1b.

[†]Infectivity measurement was determined in the Scrapie Cell End Point Assay using PK1/11 cells. PrP content was determined by ELISA. Data are reported for RML prion purification from 10 % (w/v) RML-infected CD1 brain homogenate. The level of infectivity and PrP content of the various fractions is expressed as a percentage (mean ± SEM, n = 3 preparations) of that present in the starting volume of 10 % (w/v) brain homogenate.

[‡]10 % (w/v) RML brain homogenate has a prion infectivity titre of $10^{6.5}$ tissue culture infectious units (TCIU)/ml in PK1/11 cells (Refs. 20, 21) or $10^{7.7}$ TCIU/ml when measured in PK1/2 cells (Ref 19) and a prion titre (mean ± SD, n=6) of $10^{7.3\pm0.5}$ intracerebral LD_{50} units/ml determined by endpoint titration in Tg20 mice. The PrP concentration of 10% (w/v) RML brain homogenate was determined as 5.5 ± 0.1 µg ml^{-1} (mean ± SD, n=3 determinations) by ELISA.

[§]Treatment of 10 % (w/v) RML brain homogenate with 100 µg ml^{-1} pronase E for 30 min leads to an apparent increase in prion infectivity titre (Ref. 21). The physical basis for this increase remains unexplained, but may reflect changes in PrP aggregate size with concomitant alteration of specific prion infectivity or increased bioavailability of prions in cell culture following removal of other proteins.

[‖] The method produces a recovery of ~10% of both the starting prion infectivity and PrP content in the P4 pellet fraction. Resuspension of the P4 pellet fraction in one tenth of the volume of the starting 10% (w/v) brain homogenate from which it was derived therefore produces a sample whose prion titre and PrP concentration is congruent with that of the starting 10% (w/v) brain homogenate.

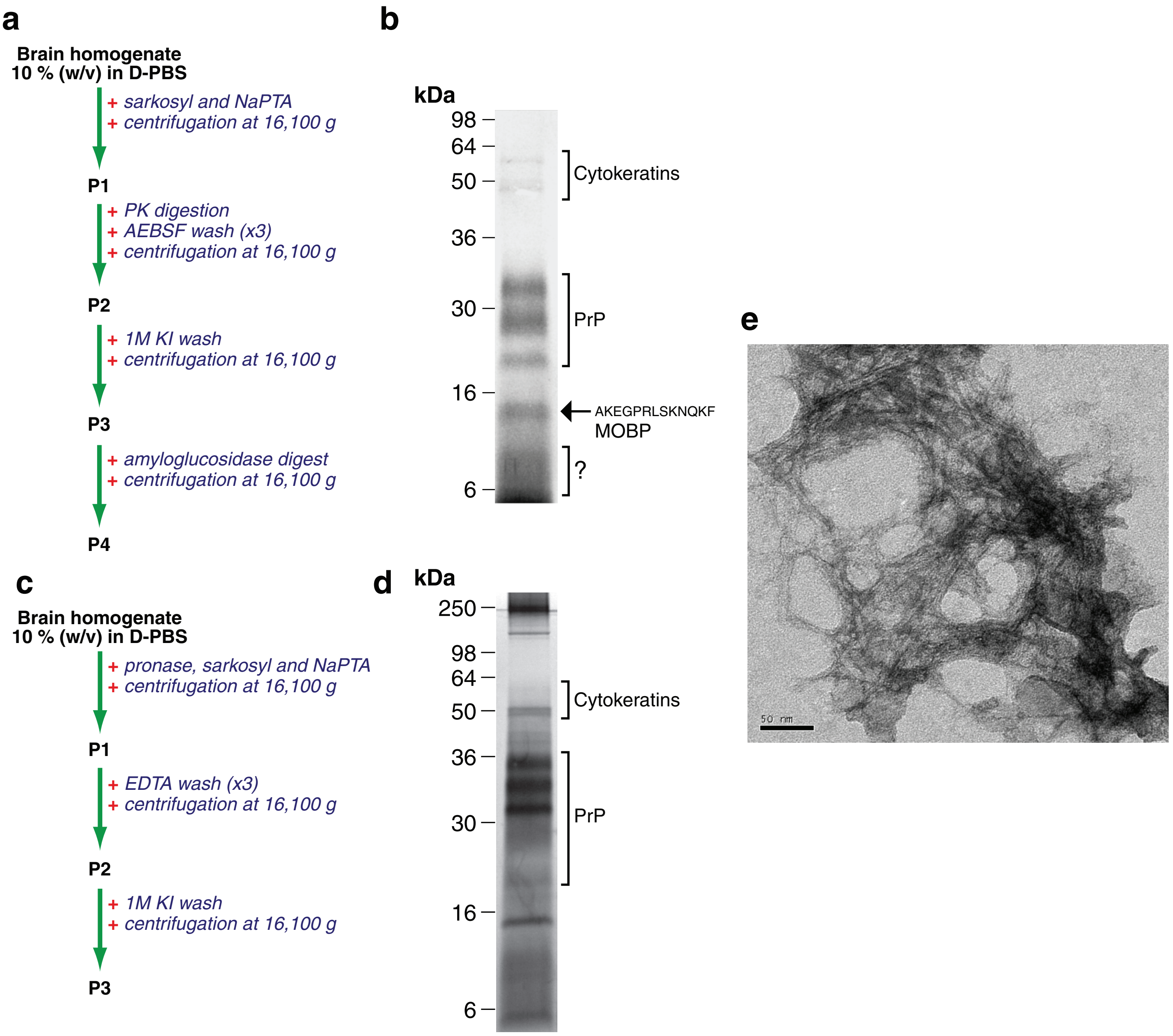

Figure S1. Identification of proteins that co-purify with mouse RML prions. We used proteinase K (PK) or pronase E digestion, NaPTA precipitation and salt (KI) extraction to generate crude insoluble RML prion preparations from the brains of terminally-affected CD1 mice. Full details of these methods can be provided on request. Flow charts showing the steps in these purification schemes (P denotes pellet fraction) are shown in panels **a** and **c** with representative silver-stained 16% SDS-PAGE gels of the final pellet fractions (the equivalent of ~200 μl of 10% (w/v) brain homogenate) shown in panels **b** and **d**. Typically the final pellet fractions contained between 10-20% of the input RML prion infectivity and were enriched for pathological prion protein (PrP) together with reproducible patterns of co-purifying proteins. Analyses of the final

pellet fractions by electron microscopy showed large, heterogeneous concretions of protein (stained with uranyl acetate; scale bar 50 nm) (**e**) that were often accompanied by collagen fibrils (not shown). Selected proteins that co-purified with PrP were identified by mass spectrometry (MS) and tandem mass spectrometry (MS/MS) or N-terminal amino acid sequencing by the University of Bristol Proteomics Facility service. For MS and MS/MS, SYPRO Ruby-stained protein bands were excised from 16% SDS-PAGE gels, digested with trypsin and analysed by MS or MS/MS using an Applied Biosystems Q-Star or 4700 TOF/TOF mass spectrometer, respectively. Peptide mass fingerprints were analysed using the Mascot search engine from Matrix Science. For amino acid sequencing, 16% SDS-PAGE gels were electro-blotted onto Immobilon-P_{SQ} membrane after which Coomassie blue-stained protein bands were excised and used to determine partial N-terminal amino acid sequence using an Applied Biosystems Procise 492cLC protein sequencer. The identity of PrP was confirmed by MS and amino acid sequencing, intermediate filament fingerprints (dominated by cytokeratins) were identified by MS/MS and myelin-associated oligodendrocyte basic protein (MOBP) by amino acid sequencing. Panel b shows the MOBP sequence obtained, in single letter amino acid nomenclature.

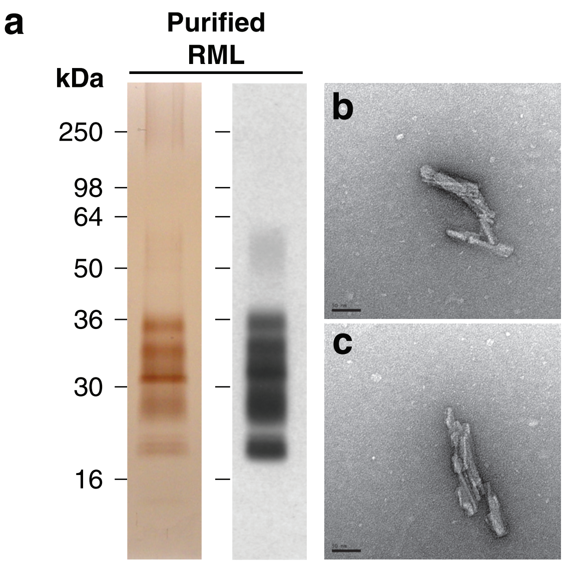

d

Dilution	Tg20 bioassay n/n_0			Tg20 bioassay Mean survival time (days)	SCEPA w/w_0	SCEPA Mean spot count
10^{-2}	6/6	6/6	6/6	69 ± 0.4	ND	-
10^{-3}	6/6	6/6	6/6	78 ± 1	ND	-
10^{-4}	6/6	6/6	4/5	87 ± 2	6/6	1199
10^{-5}	2/5	4/6	3/6	108 ± 3	6/6	292
10^{-6}	0/6	0/5	0/6	>150	8/24	223
10^{-7}	0/6	0/6	0/6	>150	0/24	0
10^{-8}	0/6	0/6	0/5	>150	0/24	0

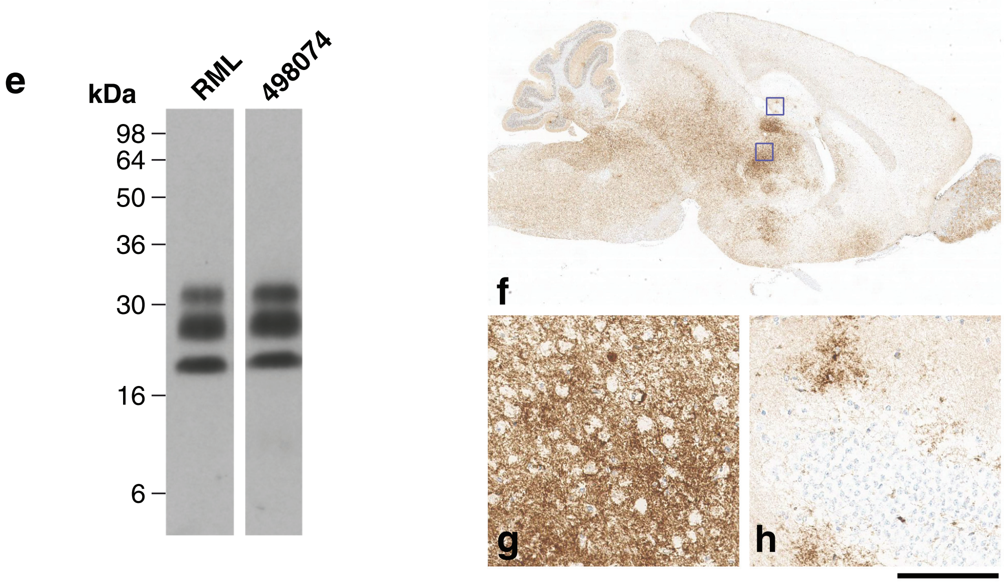

Figure S2. End-point titration of purified RML prions in Tg20 mice. We purified RML prions (without PK-digestion) from 2 ml of 10% (w/v) brain homogenate prepared from terminally-affected CD1 mice (I6200) and pooled the P4 pellet fractions in 200 µl of D-PBS containing 0.1% (w/v) sarkosyl. Immediately, a 100 µl aliquot of the purified sample was serially diluted ten-fold to concentrations of 10^{-1} to 10^{-8} in 1% (w/v) normal CD1 brain homogenate (I14041) after which these samples were stored frozen at -70°C. The remainder of the purified sample was divided into aliquots appropriate for analyses by silver-stain SDS-PAGE, PrP-immunoblotting, PrP-ELISA and electron microscopy (EM). These analyses were performed prior to mouse bioassay in order to verify that the purified RML prion preparation was entirely representative of the method. (**a**) Silver-stained 16% SDS-PAGE gel and western blot (using anti-PrP monoclonal antibody ICSM35) of the

purified RML prion sample (20 µl or 2 µl loaded, respectively). (**b,c**) EM analysis of purified RML prions, stained with uranyl acetate. Scale bar, 50 nm. Detailed inspection of grids confirmed that prion rods were the only visible protein structures in the sample. After establishing the integrity of the purified RML sample, 30 µl aliquots of each ten-fold dilution of the purified RML prions (10^{-2} to 10^{-8} concentrations) in 1% (w/v) normal CD1 mouse brain homogenate were inoculated intracerebrally into three groups of six Tg20 mice. Additionally, the same samples were used to measure prion infectivity titre using the Scrapie Cell Endpoint Assay (SCEPA). (**d**) Tg20 mouse bioassay reports the total number of mice with clinical prion disease (n) versus the total number of inoculated mice (n_0) for each group and each dilution of the purified RML prions. Mean survival time (± SEM) reports the interval from inoculation to when mice were culled with clinical prion disease in days. SCEPA reports the number of prion infected wells (w) versus the total number of inoculated wells (w_0) for each of the dilutions of the purified RML prions. Mean spot count provides evidence of prion infected wells, and typically the minimum threshold for a positive sample is 20 spots per well. In order to confirm clinical prion disease in Tg20 mice, brains from affected mice in the end-point groups (10^{-5} dilution) were analysed for abnormal PrP by immunohistochemistry (IHC) and western blot. All mice in these groups that were scored positive for clinical prion disease showed evidence of prion infection in their brain. Panels **e-h** show the results of these analyses for one Tg20 mouse (ID 498074) that was culled with clinical prion disease 89 days after inoculation with purified RML prions at 10^{-5} concentration. (**e**) Western blot (using anti-PrP monoclonal antibody ICSM35) showing PK-digested 10% (w/v) brain homogenate from either a positive control preparation, I13100 (RML) or Tg20 mouse 498074. (**f**) IHC analysis of a sagittal brain section from Tg20 mouse 498074 using anti-PrP monoclonal antibody ICSM35. The areas in the boxes are shown at higher magnification in panels (**g**), the thalamus, and (**h**), the hippocampus. Scale bars, (**f**) 2.3 mm, (**g**,**h**) 100 µm. Specific infectivity of the purified RML prion sample was calculated using the Reed Muench formula (Ref. 44). The PrP content of the isolated

200 µl purified RML prion sample was 6.9 µg/ml giving a mean specific infectivity (±SD) of purified RML prions of $10^{8.8 \pm 0.2}$ intra-cerebral LD_{50}/mg PrP.

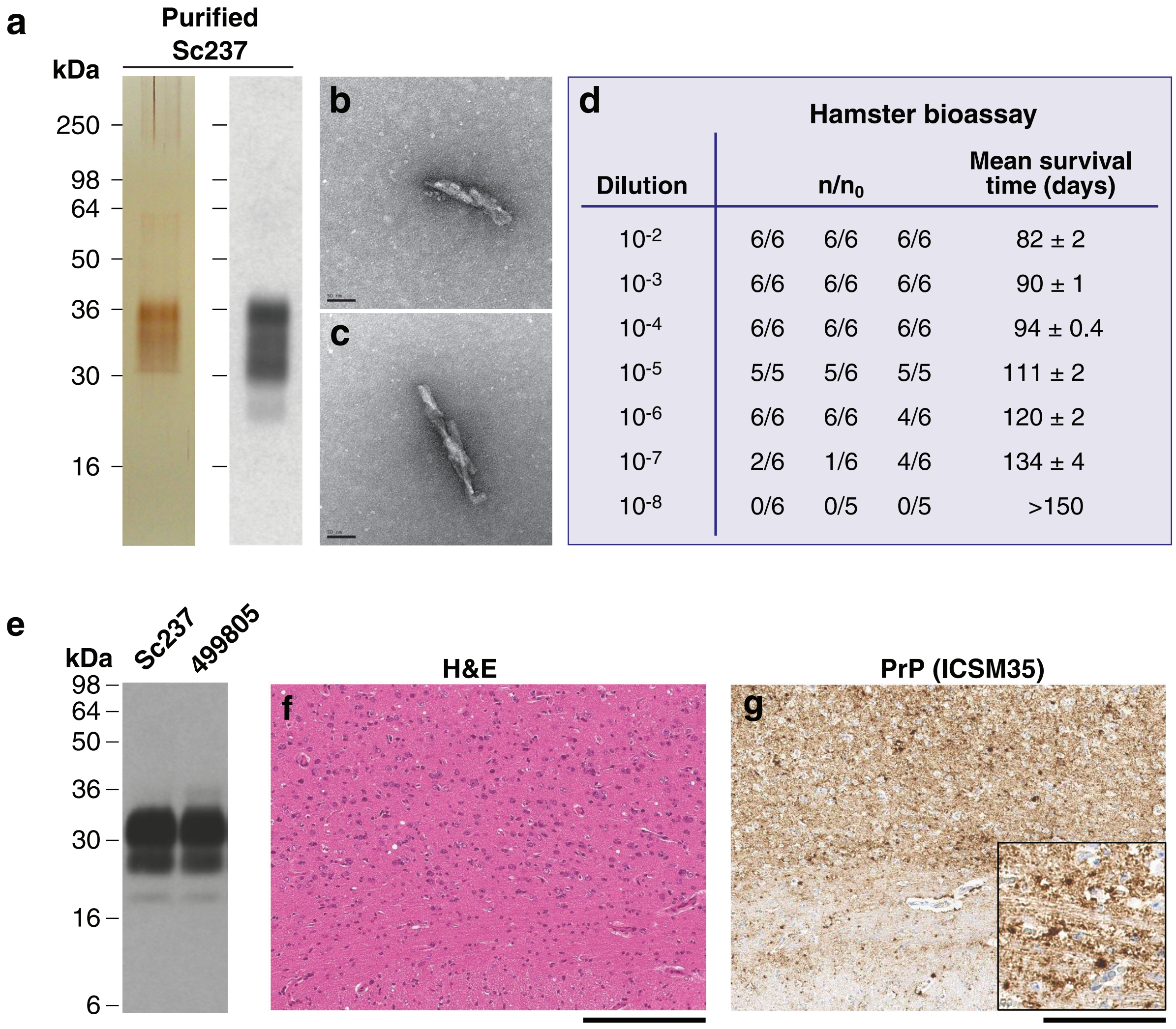

Dilution	n/n_0			Mean survival time (days)
10^{-2}	6/6	6/6	6/6	82 ± 2
10^{-3}	6/6	6/6	6/6	90 ± 1
10^{-4}	6/6	6/6	6/6	94 ± 0.4
10^{-5}	5/5	5/6	5/5	111 ± 2
10^{-6}	6/6	6/6	4/6	120 ± 2
10^{-7}	2/6	1/6	4/6	134 ± 4
10^{-8}	0/6	0/5	0/5	>150

Figure S3. End-point titration of purified hamster Sc237 prions in hamsters. We purified hamster Sc237 prions (without PK-digestion) from 2 ml of 10% (w/v) brain homogenate prepared from terminally-affected hamsters (I9200) and pooled the P4 pellet fractions in 200 µl of D-PBS containing 0.1% (w/v) sarkosyl. Immediately, a 100 µl aliquot of the purified sample was serially diluted ten-fold to concentrations of 10^{-1} to 10^{-8} in 1% (w/v) normal hamster brain homogenate (I16170) after which these samples were stored frozen at -70°C. The remainder of the purified sample was divided into aliquots appropriate for analyses by silver-stain SDS-PAGE, PrP-immunoblotting, PrP-ELISA and electron microscopy (EM). These analyses were performed prior to hamster bioassay in order to verify that the purified Sc237 prion preparation were entirely representative of the method. (**a**) Silver-stained 16% SDS-PAGE gel and western blot (using anti-

PrP monoclonal antibody ICSM35) of the purified Sc237 prion sample (20 µl or 2 µl loaded, respectively). (**b,c**) EM analysis of purified Sc237 prions, stained with uranyl acetate. Scale bar, 50 nm. Detailed inspection of grids confirmed that prion rods were the only visible protein structures in the sample. Having established the integrity of the purified Sc237 prion sample, 30 µl aliquots of each ten-fold dilution of the purified Sc237 prions (10^{-2} to 10^{-8} concentrations) in 1% (w/v) normal hamster brain homogenate were inoculated intracerebrally into three groups of six Syrian hamsters. (**d**) Hamster bioassay reports the total number of hamsters with clinical prion disease (n) versus the total number of inoculated hamsters (n_0) for each group and each dilution of the purified Sc237 prions. Mean survival time (± SEM) reports the interval from inoculation to when hamsters were culled with clinical prion disease in days. In order to confirm clinical prion disease, brains from affected hamsters in the end-point groups (10^{-7} dilution) were analysed for abnormal PrP by immunohistochemistry (IHC) and western blot. All hamsters in these groups that were scored positive for clinical prion disease showed evidence of prion infection in their brain. Panels **e-g** show the results of these analyses for one hamster (ID 499805) that was culled with clinical prion disease 135 days after inoculation with purified Sc237 prions at 10^{-7} concentration. (**e**) Western blot (using anti-PrP monoclonal antibody ICSM35) showing PK-digested 10% (w/v) brain homogenate from either the starting preparation, I9200, (Sc237) or hamster 499805. (**f**) Haematoxylin and eosin (H&E) staining, or (**g**) IHC with anti-PrP monoclonal antibody ICSM35 (PrP), in the brain (cortical region) of hamster 499805. Scale bar, main panels 200 µm, inset 50 µm. Specific infectivity of the purified Sc237 prion sample was calculated using the Reed Muench formula (Ref. 44). The PrP content of the isolated 200 µl purified Sc237 prion sample was 8.3 µg/ml giving a mean specific infectivity (±SD) of purified Sc237 prions of $10^{10.5 \pm 0.3}$ intra-cerebral LD_{50}/mg PrP.

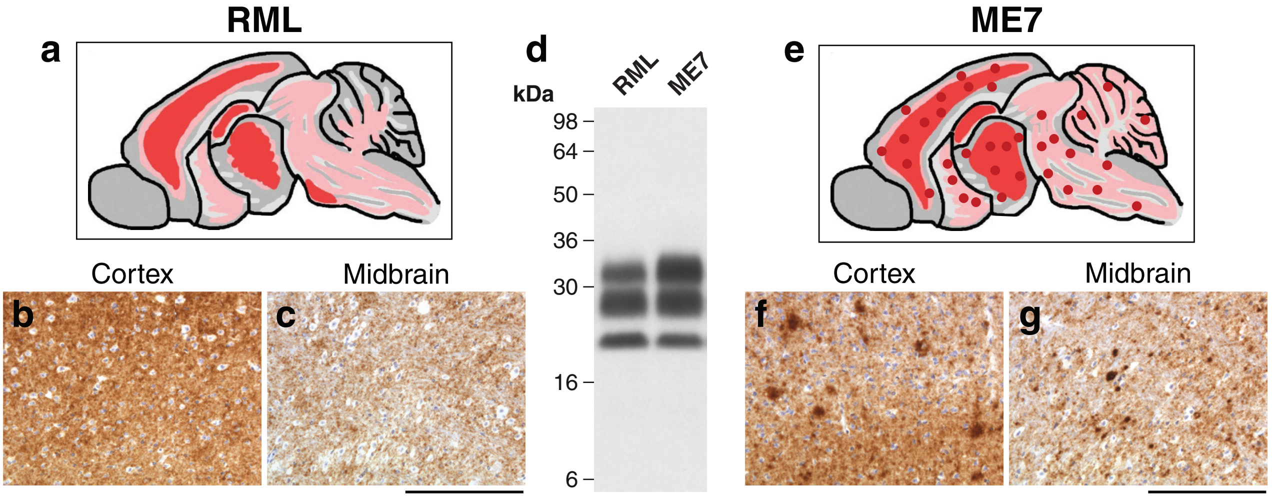

Figure S4. Propagation of ME7 and RML prion strains in C57Bl/6 mice. We inoculated groups of C57Bl/6 mice intracerebrally with either ME7 or RML prions (1% (w/v) brain homogenate) and used 30 whole brains from terminally-affected recipients to generate two pools of 10% (w/v) brain homogenate (designated I14050 ME7 and I14051 RML). The mean incubation period (± SEM) for the 30 mice contributing brain to each homogenate pool was 148 ± 1 days (n = 30) for RML and 160 ± 1 days (n = 30) for ME7. Both homogenates reported approximate titres of $10^{5.0}$ tissue culture infectious units / ml in the Scrapie Cell End Point Assay using LD9 cells. Brains from other terminally affected C57Bl/6 mice from the same inoculated groups were analysed by immunohistochemistry (IHC) in order to confirm the occurrence of specific patterns of abnormal PrP deposition that distinguish the ME7 and RML prion strains. Panels **a** (RML) and **e** (ME7) show schematic representations of abnormal PrP deposition throughout the brain (pink shading, moderate PrP deposition, red shading, intense PrP deposition, red dots, PrP microplaques). Panels **b**,**c** (RML) and **f**,**g** (ME7) show representative IHC staining patterns using anti-PrP monoclonal antibody ICSM35 in the cortex and midbrain. Scale bar, 160 μm. (**d**) Western blot (using anti-PrP monoclonal antibody ICSM35) showing PK-digested 10% (w/v) brain homogenates I14051 (RML) or I14050 (ME7). The relative proportion of di-, mono-, and non-glycosylated PrP glycoforms (%) in these PK-digested samples was, 28, 44, 28 for RML and 44, 37, 19 for ME7.

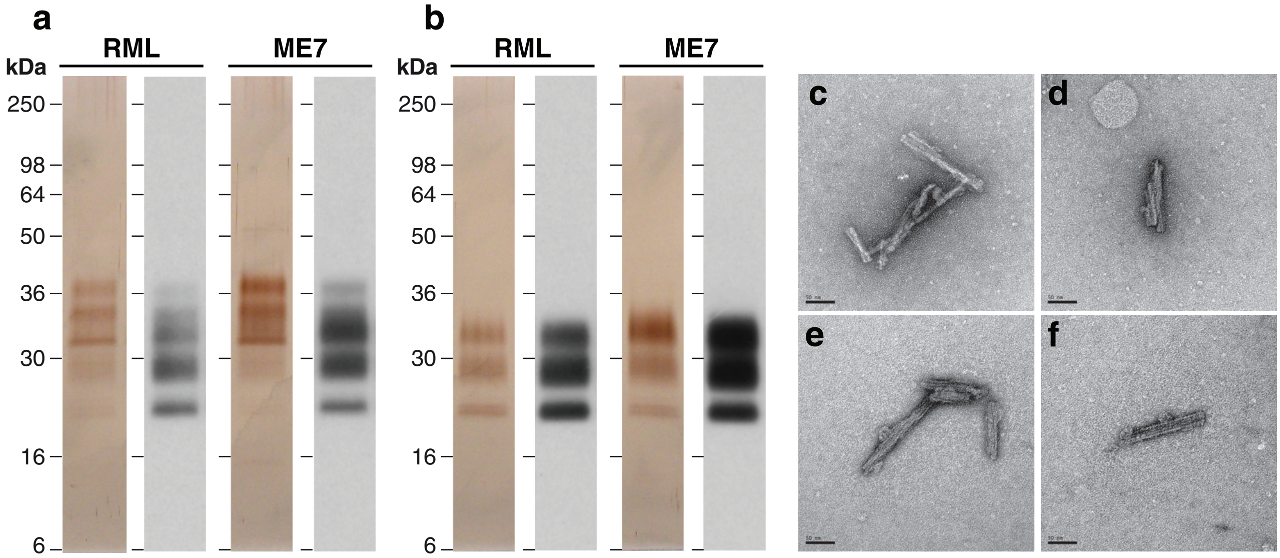

g

Inoculum		n/n_0	Mean survival time (days)
RML	Brain homogenate	13/13	163 ± 2
	Purified prions	15/15	165 ± 1
ME7	Brain homogenate	15/15	169 ± 1
	Purified prions	13/13	169 ± 1

Figure S5. Transmission of native and purified RML and ME7 prions to C57Bl/6 mice. We purified RML or ME7 prions from 2.4 ml of 10% (w/v) brain homogenates I14050 (ME7) and I14051 (RML) and pooled the respective unwashed P2 pellet fractions in 240 µl of D-PBS containing 0.1% (w/v) sarkosyl after which the samples were frozen at -70°C. Subsequently, 80 µl of the P2 samples were PK-digested and washed and the respective PK-digested P4 pellet fractions pooled in 80 µl 0.1% (w/v) sarkosyl and frozen at -70°C. The remaining 160 µl aliquots of the RML and ME7 P2 samples were washed without PK-digestion and the respective P4 pellet fractions pooled in 160 µl of D-PBS containing 0.1% (w/v) sarkosyl. Aliquots of these non-PK-digested P4 samples were serially diluted ten-fold to concentrations of 10^{-1} and 10^{-2} in 1% (w/v) normal C57Bl/6 brain homogenate (I16187) after which these samples were stored frozen at -70°C. Other aliquots of the non-PK-digested P4 RML and ME7 samples were dispensed appropriately for analyses by silver-stain SDS-PAGE, PrP-immunoblotting, PrP-ELISA and electron microscopy (EM). These analyses were performed prior to inoculating mice in order to verify that the purified

RML and ME7 prion preparations were entirely representative of the method. (**a,b**) Silver-stained 16% SDS-PAGE gels and western blots (using anti-PrP monoclonal antibody ICSM35) of the P4 RML and ME7 prion samples purified without (**a**) or with (**b**) PK-digestion (20 µl or 2 µl of purified sample was loaded per lane for silver stain or western blot, respectively). The relative proportion of di-, mono-, and non-glycosylated PrP glycoforms (%) in the P4 PK-digested samples was 27, 48, 25 for RML and 49, 36, 15 for ME7. (**c-f**) EM analysis of non-PK-digested P4 samples, stained with uranyl acetate (**c**,**d**) RML, (**e**,**f**) ME7. Scale bar, 50 nm. Detailed inspection of grids confirmed that prion rods were the only visible protein structures in the samples. After establishing the integrity of the purified RML and ME7 samples, 30 µl aliquots of non-PK-digested P4 samples at 10^{-2} concentration in 1% (w/v) normal C57Bl/6 brain homogenate were inoculated intracerebrally into groups of fifteen C57Bl/6 mice. To enable comparison of transmission properties with native prions from the starting 10% (w/v) RML or ME7 brain homogenates (I14050 and I14051), these were similarly diluted to 10^{-2} concentration in 1% (w/v) normal C57Bl/6 brain homogenate (I16187) and 30 µl aliquots inoculated intracerebrally into groups of fifteen C57Bl/6 mice. Panel **g** shows the results from these transmissions and reports the total number of mice with clinical prion disease (n) versus the total number of inoculated mice (n_0) for each group. Mean survival time (± SEM) reports the interval from inoculation to when mice were culled with clinical prion disease in days.

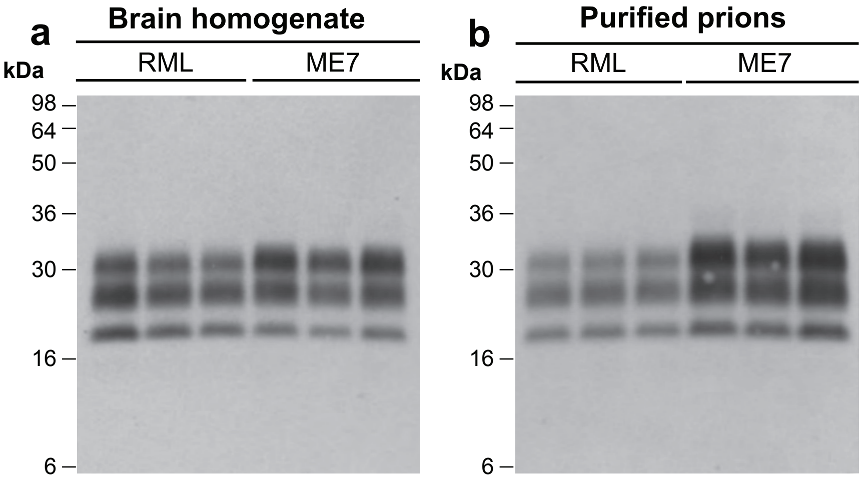

c

Inoculum		Proportion of PrP glycoforms (%) Di-	Mono-	Non-
RML	Brain homogenate	29 ± 1	46 ± 0.5	26 ± 1
	Purified prions	29 ± 0.5	46 ± 0.5	24 ± 0.5
ME7	Brain homogenate	43 ± 2	37 ± 2	20 ± 1
	Purified prions	43 ± 1	39 ± 1	18 ± 1

Figure S6. PrP glycoform ratios in C57Bl/6 mice inoculated with native or purified RML and ME7 prions. 10% (w/v) brain homogenates from terminal C57Bl/6 mice inoculated with RML or ME7 brain homogenate (native prions) or purified RML and ME7 prions (see **Figure S5**) were digested with proteinase K (PK) (50 µg/ml final protease concentration in the sample,1 h 37°C) and aliquots analysed by 16% SDS-PAGE and immunoblotting using anti-PrP monoclonal antibody ICSM35. (**a**,**b**) Representative immunoblots developed with chemiluminescent substrate showing brain samples from three mice in each inoculated group. (**a**) Mice inoculated with RML or ME7 brain homogenate. (**b**) Mice inoculated with purified RML and ME7 prions. (**c**) The relative proportion of di-, mono-, and non-glycosylated PrP glycoforms (% ± SEM; n = 5) in PK-digested brain homogenates from mice inoculated with RML or ME7 brain homogenate or purified RML and ME7 prions.
